# Wavy collector design for high-efficiency solar chimney power plants

**DOI:** 10.1038/s41598-026-49364-8

**Published:** 2026-04-28

**Authors:** Ahmed M. Elsayed, Mohamed A. Aziz, Haitham Elshimy

**Affiliations:** 1 Mechanical Engineering Department, Faculty of Engineering, Fayoum University, Fayoum, 63514 Egypt; 2 Mechanical Engineering Department, Faculty of Engineering, Suez University, Suez, Egypt; 3 Department of Mechanical and Aerospace Engineering, United Arab Emirates University, Al Ain, United Arab Emirates

**Keywords:** Solar chimney power plant, Collector geometry, Computational fluid dynamics simulation, Wavy collector, Renewable energy, Energy science and technology, Engineering

## Abstract

This study presents a computational investigation into the aerodynamic and thermal performance enhancement of a solar chimney power plant (SCPP) through the integration of wavy geometries along the collector roof. Various wavy configurations, defined by different curvature ratios (Rc/λ = 0.5 to 2.5), and wave peak amplitude (A = 0.5R to 1.5R) were assessed and compared against a conventional straight collector design. This study utilizes Computational Fluid Dynamics (CFD) simulations based on the RANS equations to predict flow behavior, pressure distribution, temperature gradients, and air velocity profiles. Mesh independence test and model validations against experimental data were conducted to ensure numerical accuracy. Results indicate that specific wavy designs, particularly at Rc/λ = 1.5 and A = R, significantly improve airflow rate, reduce pressure at the chimney inlet, and enhance temperature gain, thereby boosting the overall driving force of the system. The study concludes that incorporating optimized wavy geometries offers a promising passive strategy for improving SCPP efficiency, supporting future designs of sustainable energy systems.

## Introduction

The increasing global demand for clean and renewable energy has intensified interest in solar energy technologies that can provide sustainable electricity with minimal environmental impact^1–3^. The Solar Chimney Power Plant (SCPP) is considered a promising passive technology for converting solar energy into electricity with relatively low operational complexity and maintenance requirements. The fundamental concept and the construction of the first pilot-scale solar chimney plant in Manzanares, Spain, were reported by Haaf et al.^4^, demonstrating the feasibility of using solar-heated air to drive an updraft through a tall chimney to generate power. Subsequent studies have investigated the thermal behavior and overall performance characteristics of SCPP systems under different operating conditions, providing detailed analyses of heat transfer, airflow dynamics, and system efficiency [5 and 6]. More recently, research efforts have focused on enhancing the performance of solar chimney systems through geometric modifications and flow-control techniques within the collector and absorber regions to improve heat transfer and airflow acceleration^7–9^. The core components of a typical SCPP include a broad solar collector that heats ambient air, a vertical chimney (tower) that induces airflow upward through density reduction, and a turbine-generator system located at the chimney base to harvest energy from the moving air mass^10–12^.

In the conventional SCPP configuration, the collector, which is a transparent or translucent roof over a heat-absorbing ground surface, plays a critical role in heating the ambient air to induce natural convection^13,14^. The performance of the system heavily depends on the efficiency of thermal energy collection and the subsequent air velocity achieved at the chimney base^15–17^. As such, many studies have focused on optimizing collector dimensions, materials, and flow characteristics to enhance overall system efficiency^18–20^. Despite these efforts, one of the key challenges in conventional flat or inclined collectors is the limited enhancement in convective heat transfer, especially under low solar irradiance conditions or non-ideal wind environments^21–23^. To address this, recent research in passive solar systems has turned to surface geometry modification as a promising route for performance improvement^24,25^.

Inspired by the heat transfer benefits observed in corrugated or wavy surfaces in heat exchangers and solar air heaters^26–28^, this study proposes the integration of a wavy-shaped solar collector into the SCPP design. Wavy surfaces introduce controlled turbulence and increase the effective surface area, both of which are known to improve convective heat transfer without requiring external power input^29–31^. In addition, wavy geometries can affect solar radiation absorption profiles, potentially leading to higher localized temperatures and improved buoyancy-driven flow^32^.

Recent advancements in solar chimney design have increasingly focused on collector optimization to enhance air heating and flow acceleration. A noteworthy experimental study by El Hadji et al.^33^ studied the impact of solar radiation concentration on a solar chimney collector (SCCC) designed for natural ventilation in buildings. The study compared solar chimneys equipped with and without solar reflectors. The results demonstrated that the addition of reflectors significantly increased the collector air temperature and inlet velocity, resulting in improved thermal comfort and natural ventilation performance. Yapıcı et al.^34^ performed a numerical investigation using CFD simulations to assess how various geometric parameters influence SCPP performance. Their analysis revealed that chimney height, diameter, and collector dimensions substantially affect system efficiency. Among different configurations, diverging chimney geometry yielded the best overall performance.

Further improvements in collector design have been explored by Rezaei et al.^35^, who developed a configuration featuring metallic tubes suspended from the collector canopy to intensify solar absorption. Through combined experimental and CFD analysis, the study demonstrated that this modification could enhance air temperature and flow velocity, boosting the efficiency of the collector by up to 34% compared to traditional designs. Similarly, Nasraoui et al.^36^ investigated alternative collector configurations using validated CFD models. The study compared a traditional collector with double-pass parallel flow and double-pass counter-flow designs. Findings showed that the counter-flow double-pass collector significantly improved thermal efficiency, achieving an efficiency increase of 28% over the conventional setup. Mehdipour et al.^37^ have conducted an experimental study using controlled indoor setups, which revealed that traditional chimney geometries exhibit poor thermal and hydraulic performance, largely due to the low efficiency of standard collectors. By redesigning the collector from a circular to a square shape, significant enhancements were achieved.

Recent studies have emphasized the importance of passive cooling solutions to counteract the efficiency losses experienced by solar photovoltaic panels in high-temperature regions. One investigation employed CFD to evaluate the effect of fin count on surface temperature reduction and airflow within a hybrid solar chimney collector system^38^. The results indicated that while increasing the number of fins improved cooling efficiency, further additions led to diminished returns due to increased airflow resistance. Koonsrisuk^39^ conducted a second-law thermodynamic analysis to compare the efficiency and entropy generation of both the traditional (CSCPP) and sloped (SSCPP) designs of Solar Chimney Power Plants. Results revealed that system height plays a key role in enhancing performance for both configurations, indicating that sloped collector designs can offer thermodynamic advantages over conventional layouts. Hassan et al.^40^ conducted a numerical study to examine how variations in collector slope and chimney divergence angle influence the thermal and flow performance of a solar chimney power plant. Results showed that increasing the collector slope enhances both temperature and airflow velocity due to improved heat transfer.

Benettayeb et al.^41^ conducted a computational study to evaluate the effect of a triangular sloped absorber surface on the performance of a solar chimney system. The researchers used CFD simulations to assess how varying the central height and absorber slope radius influences thermal and aerodynamic performance. The results revealed a 26% increase in maximum airflow velocity at the central height. This geometric optimization resulted in a 34% increase in power output. Ahmad et al.^42^ introduced an approach to enhance solar chimney efficiency by incorporating Tangential Partition Walls (TPWs) within the collector. Using a CFD model, the study examined the influence of TPWs on airflow behavior and thermal performance. The swirling motion induced by TPWs significantly enhanced the uniformity and velocity of the air entering the chimney. Rahimi-Larki et al.^43^ investigated the performance of a sloped-collector solar chimney system (SCSC) subjected to ambient crosswind (ACW) using CFD simulations. Findings showed that ACW at 12 m/s could reduce output by 55%, but an optimal slope angle of 20° increased power generation by 10–35% over conventional flat collectors.

Collectively, these studies emphasize that collector geometry plays a pivotal role in indicating the thermal and aerodynamic performance of solar chimney systems. Building on these findings, the present work proposes the use of a wavy (sinusoidal) floor geometry in the collector region to promote turbulence, increase surface area, and enhance heat transfer rates without the need for active components. This study aims to fill this gap by conducting a numerical investigation into the thermal and fluid dynamic behavior of an SCPP equipped with a sinusoidal wavy collector surface. Previous SCPP studies have mainly focused on conventional flat collectors or other modifications such as sloped collectors, double-pass collectors, or curved guide vane configurations to improve airflow and thermal efficiency. However, the potential aerodynamic and thermal effects of wavy collector geometry have not been sufficiently explored. The present work advances the current state of research by introducing a wavy collector profile that modifies the air flow path inside the collector, increasing flow mixing and improving solar heat absorption distribution. This geometric modification aims to enhance the pressure gradient driving the updraft flow toward the chimney, which can lead to improved mass flow rate and power output compared with traditional collector configurations. Therefore, the proposed design provides a new passive approach to improving SCPP performance through geometric optimization of the collector, contributing to the ongoing development of more efficient solar chimney systems. The objective is to assess how variations in wave amplitude and wavelength influence airflow patterns, temperature distributions, and power generation potential compared to traditional flat designs. By integrating CFD tools and parametric modeling, this work seeks to identify optimal wave geometries that maximize performance while maintaining structural feasibility. Table 1 summarizes various studies that explored modifications to the collector geometry in SCPPs. These modifications are aimed at enhancing thermal performance, airflow, and overall system efficiency.

**Table 1:** Collector geometry modifications comparative in solar chimney power plants.

Study	Collector geometry modification	Methodology	Performance improvement	Key findings
Koonsrisuk^39^	Sloped collector vs. conventional flat design	Thermodynamic second-law analysis	Increase second-law efficiency, decrease entropy generation	SSCPP exhibited better thermodynamic performance for specific sizes; optimal collector height critical for minimizing energy degradation
Hassan et al.^40^	Collector slope (2°–10°) and chimney diverging angle (1°–5°)	CFD simulation	Increase air velocity, pressure differential, and heat transfer	An optimal diverging angle (3°) and moderate slope (6°–8°) improved system efficiency while avoiding recirculation losses
Yapıcı et al.^34^	Variation in chimney and collector dimensions	CFD simulation (parametric study)	Increase power output (geometry-dependent)	Diverging chimney and optimal collector size improved overall system efficiency
Nasraoui et al.^36^	Double-pass collector (parallel & counter flow)	CFD (validated)	Increase Efficiency by 28% (counter-flow)	Counter-flow design optimized heat exchange, showing clear advantage over conventional collectors
Mehdipour et al. (2020)^37^	Diverging collector with trapezoidal cross-section	CFD simulation	Increase flow uniformity and system efficiency	Modifying collector to a trapezoidal shape enhanced airflow distribution and improved pressure-driven output
El Hadji et al.^33^	Addition of reflectors to a flat collector	Experimental (2 × 2 × 2 m test setup)	Increase air temperature and velocity	Reflectors enhanced solar radiation concentration, improving natural ventilation and thermal comfort
Aziz and Elsayed^29^	Variations in collector inclination and height–diameter ratio	CFD + Experimental Validation	Increase air velocity, temp gradient, system power	Thermo-fluid analysis showed that chimney geometry, particularly collector slope and chimney-to-collector ratio, strongly influences thermal efficiency and flow development
Rezaei et al.^35^	Hanging metallic tubes from collector canopy	CFD + Experimental validation	Increase Nu and Velocity	Collector modification significantly enhanced heat transfer and airflow, improving SCPP output
Genge et al.^38^	Hybrid solar chimney-collector system equipped with wavy cooling fins	CFD + Experimental validation	Decrease surface temp by 14.1°C, increase cooling efficiency	Optimized wavy fins reduced PV temperature and enhanced passive cooling in hot climates
Elsayed et al.^22^	Curved-guide vane configurations within the collector	Experimental validation + CFD Optimization study	Increase collector efficiency; increase airflow guidance	Curved vanes improved thermal stratification and directed airflow, leading to better overall system performance
Benettayeb et al.^41^	Triangular sloped absorber (central height + slope radius)	CFD	Increase maximum air velocity by 26%, increase power output by 34%	Optimized sloped absorber dimensions significantly improved flow and power output in Manzanares-based design
Ahmad et al.^42^	Tangential Partition Walls (TPWs) in collector	CFD + Experimental validation	Increase kinetic energy by 350%, increase air velocity & uniformity	Swirling flow induced by TPWs enhanced updraft strength and reduced recirculation, boosting SC output
Rahimi-Larki et al.^43^	Sloped collector (20°–30°) under ambient crosswind	CFD simulation	Increase power by 10–35%; Increase recovery of 16% loss at 12 m/s crosswind	Optimal slope boosts performance and mitigates crosswind impact; adaptive geometry is essential for large-scale SCSC
Current study	Wavy-shaped solar collector with multiple sinusoidal geometries	CFD simulation	Increased airflow velocity, enhanced pressure gradient, improved thermal buoyancy	Sinusoidal collector geometry demonstrated a consistent performance enhancement, with optimum curvature leading to higher energy conversion efficiency

## Methodology

### Geometric model

**Fig. 1:**
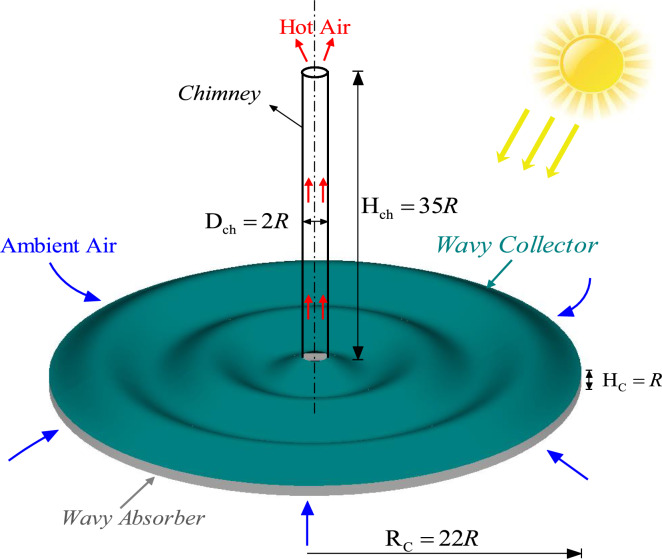
Wavy collector overall geometry definition.

The geometric parameters of the solar chimney power plant (SCPP) used in the present study were selected based on commonly reported configurations in previous experimental and numerical investigations to ensure realistic operating conditions and meaningful comparison with the literature [8, 22, 36, and 44]. Figure 1 presents the overall geometry of the selected solar chimney, which is defined by a straight chimney of radius R = 0.084 m, height H_ch_ = 35R, a wavy collector of radius R_C_ = 22R, and a collector height H_C_ = R. The wavy collector design depicted in Fig. 1 follows a periodic cosine-shaped profile, which can be mathematically expressed as

1 $$y=A\mathrm{cos}\left(\frac{2\pi }{\lambda }r\right)$$

**Fig. 2:**
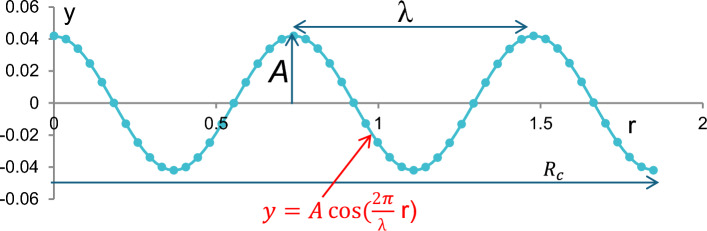
Definition of Wavy Collector Surface Geometry.

In Eq. (1), *y* represents the vertical displacement of the collector surface at any point r, which lies along the axial (horizontal) direction of the collector. Parameter *A* denotes the amplitude of the wave in the range of 0.5R:1.5R meters, while λ represents the wavelength, or the distance between two successive wave peaks in the range of* R*_*c*_/0.5: *R*_*c*_/2.5 m. Figure 2 illustrates the mathematical formulation of the wavy collector surface applied in the current solar chimney power plant design.

The selected curvature ratios and wave amplitudes were chosen to systematically evaluate the influence of collector surface waviness on the thermo-fluid behavior within the solar chimney system. The investigated range was defined to cover moderate geometric variations that are sufficient to modify the airflow path and enhance air surface interaction without causing excessive distortion of the collector structure or disrupting the main buoyancy-driven flow toward the chimney. From an engineering perspective, the selected amplitudes remain relatively small compared with the overall collector radius, ensuring that the structural integrity and overall configuration of the collector are maintained. Moreover, such wavy geometries can be practically implemented using segmented or modular collector panels, which are commonly employed in large-scale solar thermal systems. Therefore, the selected parameter range represents a realistic balance between aerodynamic performance evaluation and potential structural feasibility for larger-scale solar chimney applications.

**Fig. 3:**
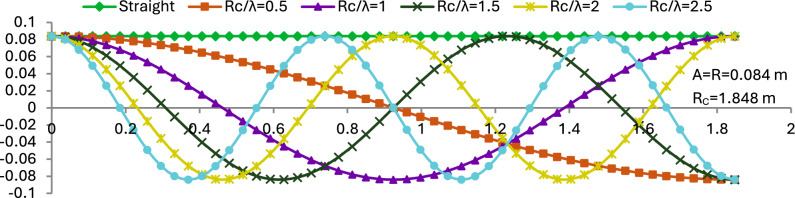
Two dimensions’ different shapes of the wavy collector compared to the straight collector.

Figure 3 illustrates the two-dimensional profiles of various wavy collector geometries compared with a conventional straight collector surface. The straight collector, shown as a horizontal line, serves as a baseline with no surface modulation. In contrast, the wavy collectors follow a cosine wave profile. Each curve in the figure corresponds to a different ratio of total collector length R_C_ to wavelength λ, including values of 0.5, 1.0, 1.5, 2.0, and 2.5. As the R_C_/λ ratio increases, the number of wave cycles across the collector length increases, resulting in more tightly packed undulations. For instance, the profile with R_C_ /λ = 0.5 shows a broad, single wave, while R_C_/λ = 2.5 displays multiple, shorter wave cycles within the same horizontal extent.

**Fig. 4:**
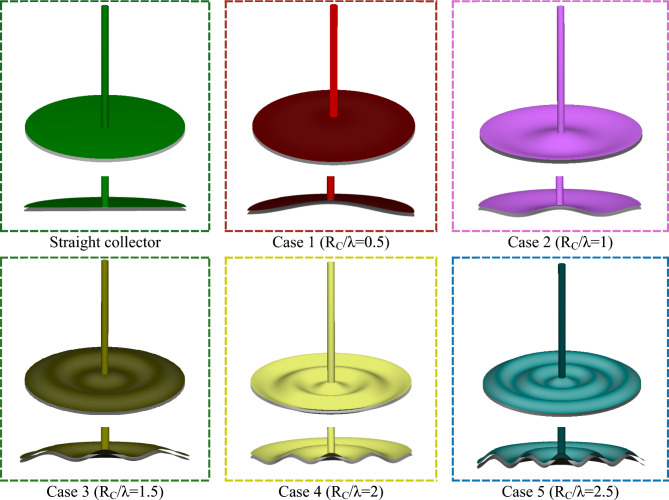
Different shapes of wavy collector solar chimney compared to a straight collector.

Figure 4 presents a detailed comparison of different three-dimensional collector geometries used in solar chimney power plants, highlighting the transition from a conventional straight collector to various wavy collector configurations. The top views show the radial symmetry of these waveforms, while the side views emphasize the vertical amplitude variations introduced in each case.

### Governing equations

The core equations that govern fluid flow form the foundation for analyzing fluid dynamics. In the case of an SCPP, the internal flow is predominantly buoyancy-driven and exhibits significant turbulence. As a result, the governing equations must be adapted to account for these characteristics, ensuring both accuracy and practicality when using CFD tools. The mathematical model is thus characterized by these modified governing equations. The continuity equation,

2 $$\frac{\partial \rho }{\partial t}+\frac{1}{r}\frac{\partial (r\rho {u}_{r})}{\partial r}+\frac{1}{r}\frac{\partial (\rho {u}_{\theta })}{\partial \theta }+\frac{\partial (\rho {u}_{z})}{\partial z}=0$$

Navier–Stokes equation,

3 $$\begin{aligned} & \rho \left( {\frac{{\partial u_{r} }}{\partial t} + u_{r} \frac{{\partial u_{r} }}{\partial r} + \frac{{u_{\theta } }}{r}\frac{{\partial u_{r} }}{\partial \theta } + u_{z} \frac{{\partial u_{r} }}{\partial z} - \frac{{u_{\theta }^{2} }}{r}} \right) \\ & \quad = \rho g_{r} - \frac{\partial p}{{\partial r}} + \mu \left[ {\frac{{\partial^{2} u_{r} }}{{\partial r^{2} }} + \frac{1}{r}\frac{{\partial u_{r} }}{\partial r} + \frac{1}{{r^{2} }}\frac{{\partial^{2} u_{r} }}{{\partial \theta^{2} }} + \frac{{\partial^{2} u_{r} }}{{\partial z^{2} }} - \frac{{u_{r} }}{{r^{2} }} - \frac{2}{{r^{2} }}\frac{{\partial u_{\theta } }}{\partial \theta }} \right] \\ \end{aligned}$$

4 $$\begin{aligned} & \rho \left( {\frac{{\partial u_{\theta } }}{\partial t} + u_{r} \frac{{\partial u_{\theta } }}{\partial r} + \frac{{u_{\theta } }}{r}\frac{{\partial u_{\theta } }}{\partial \theta } + u_{z} \frac{{\partial u_{\theta } }}{\partial z} + \frac{{u_{r} u_{\theta } }}{r}} \right) \\ & \quad = \rho g_{\theta } - \frac{1}{r}\frac{\partial p}{{\partial \theta }} + \mu \left[ {\frac{{\partial^{2} u_{\theta } }}{{\partial r^{2} }} + \frac{1}{r}\frac{{\partial u_{\theta } }}{\partial r} + \frac{1}{{r^{2} }}\frac{{\partial^{2} u_{\theta } }}{{\partial \theta^{2} }} + \frac{{\partial^{2} u_{\theta } }}{{\partial z^{2} }} - \frac{{u_{\theta } }}{{r^{2} }} + \frac{2}{{r^{2} }}\frac{{\partial u_{r} }}{\partial \theta }} \right] \\ \end{aligned}$$

5 $$\begin{aligned} & \rho \left( {\frac{{\partial u_{z} }}{\partial t} + u_{r} \frac{{\partial u_{z} }}{\partial r} + \frac{{u_{\theta } }}{r}\frac{{\partial u_{z} }}{\partial \theta } + u_{z} \frac{{\partial u_{z} }}{\partial z}} \right) \\ & \quad = \rho g_{z} - \frac{\partial p}{{\partial z}} + \mu \left[ {\frac{{\partial^{2} u_{z} }}{{\partial r^{2} }} + \frac{1}{r}\frac{{\partial u_{z} }}{\partial r} + \frac{1}{{r^{2} }}\frac{{\partial^{2} u_{z} }}{{\partial \theta^{2} }} + \frac{{\partial^{2} u_{z} }}{{\partial z^{2} }}} \right] \\ \end{aligned}$$

Energy conservation equation,

6 $$\begin{aligned} & \rho c_{p} \left( {\frac{\partial T}{{\partial t}} + u_{r} \frac{\partial T}{{\partial r}} + \frac{{u_{\theta } }}{r}\frac{\partial T}{{\partial \theta }} + u_{z} \frac{\partial T}{{\partial z}}} \right) \\ & = \rho \dot{q}_{g} - \frac{1}{r}\frac{\partial }{\partial r}\left( {kr\frac{\partial T}{{\partial r}}} \right) + \frac{1}{r}\frac{\partial }{\partial \theta }\left( {\frac{k}{r}\frac{\partial T}{{\partial \theta }}} \right) + \frac{\partial }{\partial z}\left( {k\frac{\partial T}{{\partial z}}} \right) \\ & \quad + \beta T\left( {\frac{\partial p}{{\partial t}} + u_{r} \frac{\partial p}{{\partial r}} + \frac{{u_{\theta } }}{r}\frac{\partial p}{{\partial \theta }} + u_{z} \frac{\partial P}{{\partial z}}} \right) + {\Phi } \\ \end{aligned}$$

where the viscous dissipation term is Φ it represents the rate at which mechanical energy is converted into internal energy due to the fluid’s viscous resistance. Mathematically Φ is expressed as:

7 $$\begin{aligned} {\Phi } & = 2\mu \left[ {\left( {\frac{{\partial u_{r} }}{\partial r}} \right)^{2} + \left( {\frac{1}{r}\frac{{\partial u_{\theta } }}{\partial \theta } + \frac{{u_{r} }}{r}} \right)^{2} + \left( {\frac{{\partial u_{z} }}{\partial z}} \right)^{2} } \right] \\ & \quad + \mu \left[ {\left( {\frac{1}{r}\frac{{\partial u_{r} }}{\partial \theta } + \frac{{\partial u_{\theta } }}{\partial r} - \frac{{u_{\theta } }}{r}} \right)^{2} + \left( {\frac{{\partial u_{\theta } }}{\partial z} + \frac{1}{r}\frac{{\partial u_{z} }}{\partial \theta }} \right)^{2} + \left( {\frac{{\partial u_{z} }}{\partial r} + \frac{{\partial u_{r} }}{\partial z}} \right)^{2} } \right] \\ & \quad - \frac{2}{3}\mu \left( {\frac{1}{r}\frac{{\partial \left( {ru_{r} } \right)}}{\partial \theta } + \frac{1}{r}\frac{{\partial u_{\theta } }}{\partial \theta } + \frac{{\partial u_{z} }}{\partial z}} \right)^{2} \\ \end{aligned}$$

The Boussinesq equation is defined by:

8 $$\rho ={\rho }_{0}\left[1-\beta \left(T-{T}_{0}\right)\right]$$

The turbine output power is defined by:

9 $$P_{out} = \eta_{c} \cdot \eta_{ch} \cdot A_{C} \cdot G = \frac{1}{2}\dot{m} \cdot v_{ch}^{2}$$

The efficiency of collector is presented in Eq. (10)

10 $${\eta }_{c}=\frac{\dot{m}{c}_{p}\Delta T}{{A}_{c} G}$$

The system overall efficiency is defined by:

11 $$\eta_{o} = \frac{{P_{act} }}{{A_{c} G}}$$

To account for the turbulent characteristics of the flow inside the SCPP, the standard k-ε turbulence model was integrated with these equations^22,23,29^. To optimize computational resources and efficiency, the simulation assumed symmetric boundaries and steady-state flow within the SCPP. The mathematical model is thus characterized by these modified governing equations.

### Numerical solver settings

In the current study, simulations were conducted using CFDRC (CFD-ACE+), a robust multiphysics computational fluid dynamics platform well-suited for complex thermo-fluid problems, such as buoyancy-driven flows in solar chimney systems. The discretization of the governing equations was performed using the finite volume method. For spatial discretization, second-order upwind schemes were applied to both convective and diffusive terms in the momentum and energy equations, enhancing accuracy while maintaining numerical stability. The pressure–velocity coupling was handled using the SIMPLE algorithm, which is widely recognized for its stability in solving incompressible and buoyant flows. For turbulence modeling, the standard k-ε turbulence model was adopted due to its enhanced capability in capturing swirling and recirculating flows typically found at the collector-chimney junction and near wavy surfaces. Near-wall treatment used enhanced wall functions, and the mesh was refined to maintain y⁺ values close to 1 in boundary layer regions. Convergence criteria were carefully chosen to ensure solution stability and accuracy.

The modeling assumptions adopted in the present numerical study were selected to ensure an appropriate balance between physical accuracy and computational efficiency. The standard k–ε turbulence model was employed because of its robustness and wide application in simulating turbulent buoyancy-driven flows in solar chimney power plant systems. Previous numerical studies have demonstrated that this model provides reliable predictions of velocity and temperature fields in large-scale natural convection flows with reasonable computational cost^7,22,23,41^. A steady-state approach was adopted since the main objective of this study is to evaluate the aerodynamic and thermal performance of the system under stabilized operating conditions. In solar chimney systems, once solar radiation and boundary thermal conditions become relatively stable, the airflow inside the collector chimney configuration tends to approach a quasi-steady regime, making steady-state analysis suitable for capturing the representative flow behavior and performance parameters. In addition, symmetry boundary conditions were applied in the circumferential direction to exploit the geometric symmetry of the collector configuration and reduce the computational domain while maintaining the essential flow characteristics of the full system. Furthermore, the optical properties of the collector roof were specified in the model to properly represent solar radiation transmission and heat transfer processes. The transmissivity, absorptivity, and emissivity coefficients were assigned representative values commonly reported for collector materials, namely 0.9, 0.1, and 0.9, respectively.

### Boundary conditions

**Fig. 5:**
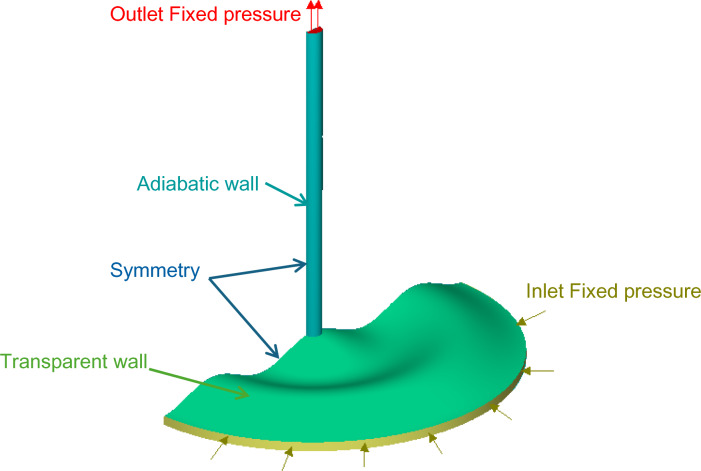
Solar chimney system boundary condition.

Figure 5 illustrates the computational domain and applied boundary conditions for an SCPP model featuring wavy collector geometry. This setup is used in CFD simulations to analyze the system’s thermo-fluid performance under solar heating conditions. At the inlet, a fixed pressure boundary condition is applied. This pressure is set to atmospheric pressure, 101,325 Pa, allowing ambient air to flow into the system with a temperature of 308 K. At the top of the chimney, a fixed pressure outlet is applied, also set to atmospheric conditions. The transparent wall, located at the upper surface of the collector, is treated as a radiation-transmitting surface. The adiabatic wall condition is applied along the vertical sides of the chimney. Symmetry boundary is implemented along the centerline to reduce the computational domain and save resources. The solution was considered converged when the residuals of the governing equations (continuity, momentum, energy, and turbulence equations) decreased below 10^–6^ for the energy equation and 10^–5^ for the remaining equations.

**Fig. 6:**
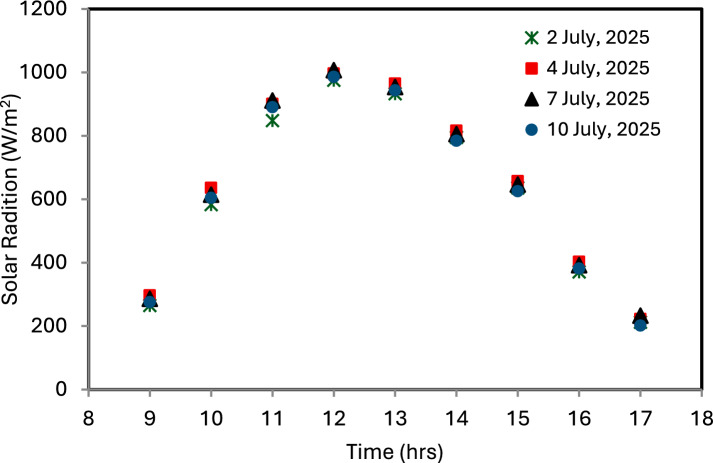
Daily changes in solar radiation levels in Giza, Egypt, during July 2025.

The measured data are represented in Fig. 6 how solar radiation levels changed in Giza, Egypt, during several days in July 2025. In the present study, the solar irradiance data for a representative month were used to evaluate the performance of the solar chimney power plant (SCPP) under typical operating conditions. The selected month corresponds to July, which represents the period of maximum solar irradiance in Giza, Egypt. The intensity shows the usual daily cycle, rising steadily after sunrise, reaching its highest values around midday (between 12:00 and 13:00), and then declining toward the late afternoon. The maximum recorded intensity ranged from about 986 to 1006 W/m^2^, which is consistent with typical summer conditions in the region. The base of the collector is modeled as a surface with a constant heat flux of 1000 W/m^2^ to simulate the effect of absorbed solar radiation. The working fluid in this model is air, treated as an ideal gas to accurately capture buoyancy effects.

In the present numerical model, the base of the collector is defined as a surface subjected to a constant heat flux of 1000 W/m^2^, representing the absorbed solar radiation per unit area. Therefore, when the collector surface is extended due to the introduction of the wavy geometry, the effective heat transfer area increases, which consequently leads to a higher total heat input into the system. This additional thermal energy contributes to increasing the air temperature within the collector, thereby strengthening the buoyancy-driven flow toward the chimney. As a result, higher airflow velocity and mass flow rates may be observed compared with the flat collector configuration. However, the improvement in system performance is not solely due to the increase in surface area; the wavy geometry also modifies the airflow path and enhances the interaction between the heated surface and the flowing air, promoting better heat transfer and flow mixing within the collector region. Consequently, the observed performance enhancement reflects the combined influence of increased effective heating area and improved flow dynamics induced by the wavy collector design.

### Mesh generation and grid independence

**Fig. 7:**
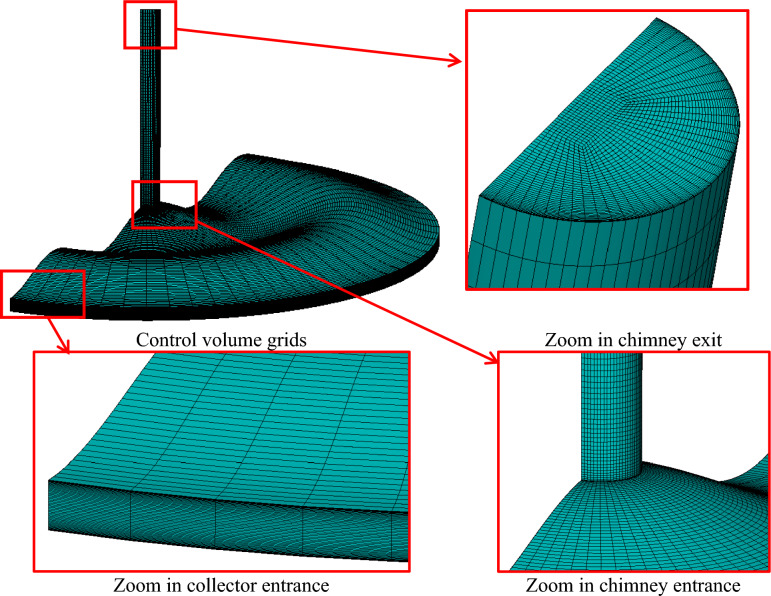
Wavy collector solar chimney structured grids.

Figure 7 illustrates the structured mesh configuration used to discretize the computational domain of a SCPP model featuring wavy collector geometry. The full 3D mesh layout, including both the collector and the vertical chimney tower, is shown. The zoomed-in views provide more detail on specific regions of interest. The structured mesh accommodates the undulating geometry without introducing highly skewed or distorted cells. The mesh near the collector’s inlet region is refined to resolve the boundary layer and incoming airflow behavior. A dense mesh is used at the connection between the chimney and the collector to resolve the sharp velocity and temperature gradients that typically develop due to buoyancy-driven acceleration of heated air. The aspect ratio was maintained at less than 5 for most cells, slightly higher near the chimney exit. The orthogonality was kept larger than 0.9 while the skewness was less than 0.25 across the mesh. The grid growth rate was less than 1.2 to ensure a smooth transition between fine and coarse zones.

**Fig. 8:**
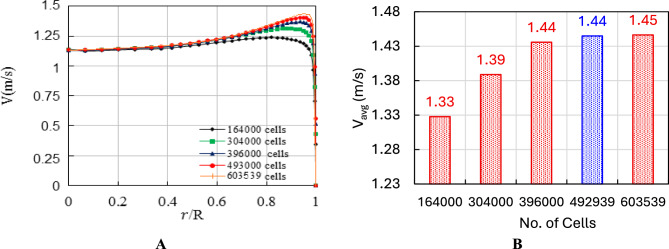
Effect of chimney number of cells on the chimney entrance (**A**) velocity profiles (m/s) and (**B**) average velocity (m/s).

Figure 8 presents the results of a mesh independence study conducted with straight collector to ensure the reliability and accuracy of the CFD simulations for the SCPP model. In Fig. 8A, the velocity profiles at the chimney entrance are plotted across the normalized radial direction (r/R) for four different mesh densities 164,000, 304,000, 396,000, and 493,000 cells. As observed, the velocity distributions begin to converge as the mesh is refined, especially beyond 396,000 cells. Figure 8B provides a bar chart summarizing the average velocity values (V_avg_) at the chimney inlet for various mesh densities. By increasing the number of cells from 164,000 to 396,000, the average velocity rises from 1.33 m/s to 1.44 m/s, signifying better resolution of the updraft flow. The average velocity values at 492,939 and 603,539 cells, both yielding approximately 1.44–1.45 m/s. This consistency in V_avg_ at the higher mesh densities confirms that mesh independence is achieved around 493,000 cells. This grid sensitivity study validates that a mesh with approximately 493,000 cells provides a good balance between computational cost and simulation accuracy.

### Validation

**Fig. 9:**
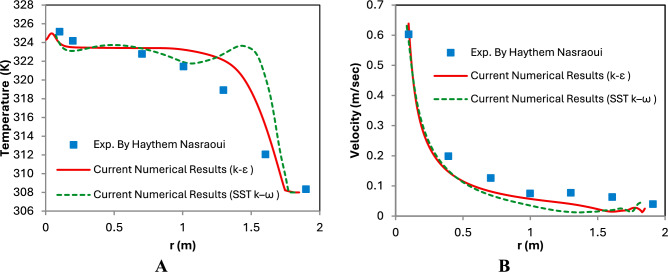
Collector mid plane (**A**) Temperature (K) (**B**) Velocity (m/sec) of experimental investigation by Haythem^44^ and the current numerical study.

The validation curves presented in Fig. 9 at the collector mid plane compare the velocity (m/s) and temperature (K) distributions obtained from the current numerical study with the experimental results reported by Haythem Nasraoui^44^. For this purpose, the validation was performed using the conventional straight (flat) collector configuration, which is consistent with the experimental setup used in the referenced study. Figure 9A shows the temperature profile along the collector’s midplane. The numerical results obtained using the standard k–ε model and the SST k–ω model show good agreement with the experimental data, with only minor deviations near the outer radius, especially in the central and mid-regions of the collector. The temperature starts around 326 K at the center and shows a gradual decline toward the edges, dropping to about 308 K. The minor deviation at the outer edge could be attributed to experimental uncertainties, edge heat losses, or boundary layer development that is more challenging to replicate precisely in CFD. Figure 9B shows the velocity distribution along the same radial direction. Both turbulence models reproduce the general behavior observed in the experimental results. The SST k–ω model slightly overpredicts the velocity in some regions, whereas the k–ε model provides a closer prediction near the outer collector region. Overall, the k–ε model, the RMSE and Mean Absolute Error (MAE) for temperature are 0.38 K and 0.5 K, respectively, while for velocity they are 0.015 m/s and 0.02 m/s. Therefore, the model is sufficiently accurate to proceed with parametric investigations and geometry optimization in SCPP studies. The analysis shows that the average percentage error for temperature is approximately 0.154%, while the average percentage error for velocity is about 3.23%, which indicates good agreement between the present simulation and the experimental measurements.

The validation of the numerical model was carried out by comparing the present simulation results with previously published experimental and numerical data to ensure the reliability and accuracy of the computational approach. In this study, the Root Mean Square Error (RMSE) was used as a quantitative metric to evaluate the deviation between the predicted results and the reference data. The RMSE is defined as,

$$RMSE=\sqrt{\frac{1}{n}\sum_{i=1}^{n}{\left({X}_{num,i}-{X}_{ref,i}\right)}^{2}}$$

where ${X}_{num,i}$ represents the numerical result obtained from the present model, ${X}_{ref,i}$ represents the corresponding value reported in the reference study, and *n* is the total number of data points used for comparison. In addition to RMSE, the percentage error was also calculated to further assess the level of agreement between the predicted and reference results. The percentage error is defined as

$$\%Error=\left|\frac{{X}_{num}-{X}_{ref}}{{X}_{ref}}\right|\times 100$$

Table 2 presents a comparison demonstrating that the predicted velocity and temperature distributions obtained from the present model show good agreement with the reference study, with relatively small RMSE values and acceptable percentage deviations. These results confirm that the adopted numerical methodology, including the governing equations, turbulence model, and boundary conditions, is capable of accurately predicting the thermo-fluid behavior inside the solar chimney system. Therefore, the validation results support the reliability of the developed numerical model for analyzing the performance of the proposed wavy collector configuration.

**Table 2:** Error analysis between present numerical results using k–ε model and reference study.

Parameter	Reference value	Present study value	Absolute error	Percentage error (%)	RMSE
Air velocity at chimney inlet (m/s)	0.62	0.6	0.02	3.23	Velocity (m/s) 0.015
Air temperature at collector outlet (K)	325.2	324.7	0.5	0.154	Temperature (K) 0.38
Mass flow rate (kg/s)	0.0351	0.0348	0.0003	0.855	0.00028

## Results and discussion

### Effect of wavy length, λ, on the Flow Field

**Fig. 10:**
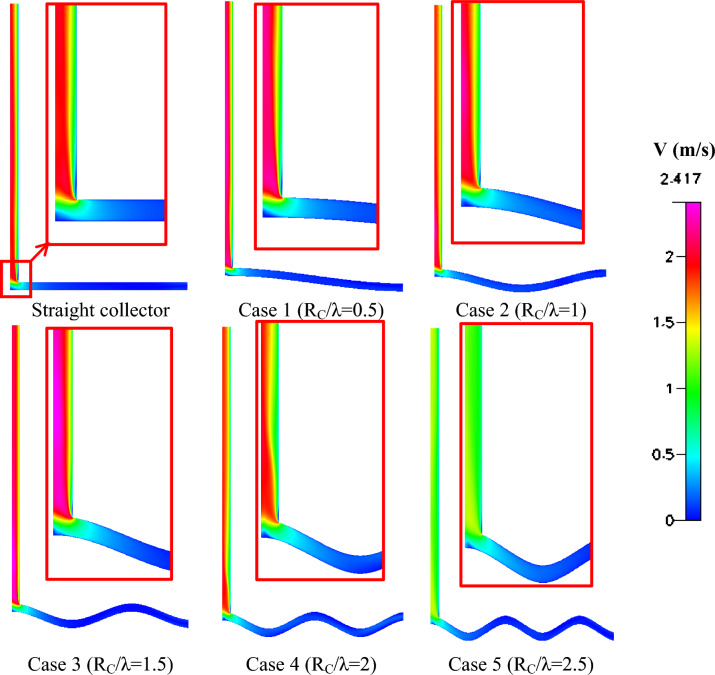
Different shapes velocity magnitude contours (m/sec) of the wavy collector solar chimney compared to the straight collector.

To assess the aerodynamic and thermal enhancements introduced by the wavy collector design, multiple CFD post-processing visualizations were conducted and compared with the baseline straight collector configuration. As the curvature increases, the size of the air path in the collector increases. The results are presented as contours for flow field variables at constant amplitude (A = R). Figure 10 shows the velocity magnitude contours (m/s), clearly illustrating the changes in flow acceleration and turbulence patterns due to the introduction of sinusoidal surface undulations. The wavy geometries exhibit enhanced velocity zones in the collector due to improved natural convection and air channeling effects along the wavy walls. As the path length increases, the velocity increases compared to the straight collector. It was observed that the highest velocity at the chimney inlet is associated with case 3, where R_c_/λ = 1.5, followed by case 4, where R_c_/λ = 2.

Among the tested cases, Rc/λ = 1.5 (Case 3) demonstrates the most favorable aerodynamic behavior. The velocity contours show a more continuous acceleration of the flow along the collector, resulting in the highest velocity magnitude near the chimney entrance. This behavior can be attributed to the moderate curvature that increases the effective surface interaction without introducing excessive flow resistance. In contrast, when the curvature becomes too large, as in Rc/λ = 2.5 (Case 5), the airflow experiences additional resistance due to the longer and more tortuous flow path, which partially offsets the benefits of enhanced heat transfer.

**Fig. 11:**
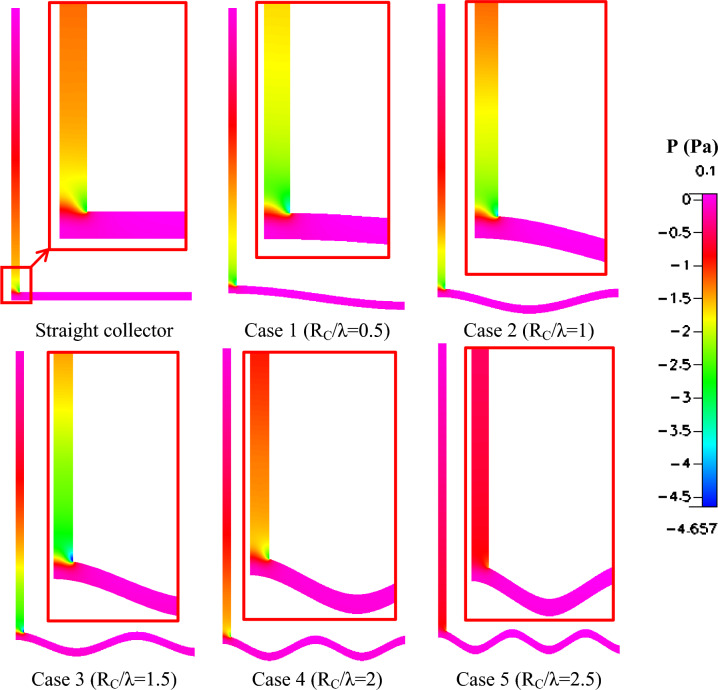
Different shapes of pressure contours (Pa) of wavy collector solar chimney compared to straight collector.

The pressure contours illustrated in Fig. 11 further confirm this behavior. In solar chimney systems, the pressure gradient between the collector inlet and the chimney base is the primary driving force for airflow. The results indicate that the wavy collector geometries produce a stronger negative pressure region near the chimney entrance compared with the straight collector. This enhanced pressure drop promotes stronger suction and increases the buoyancy-driven mass flow rate. The most pronounced pressure reduction occurs again for Rc/λ = 1.5, indicating that this geometry provides the most effective balance between flow acceleration and pressure losses.

**Fig. 12:**
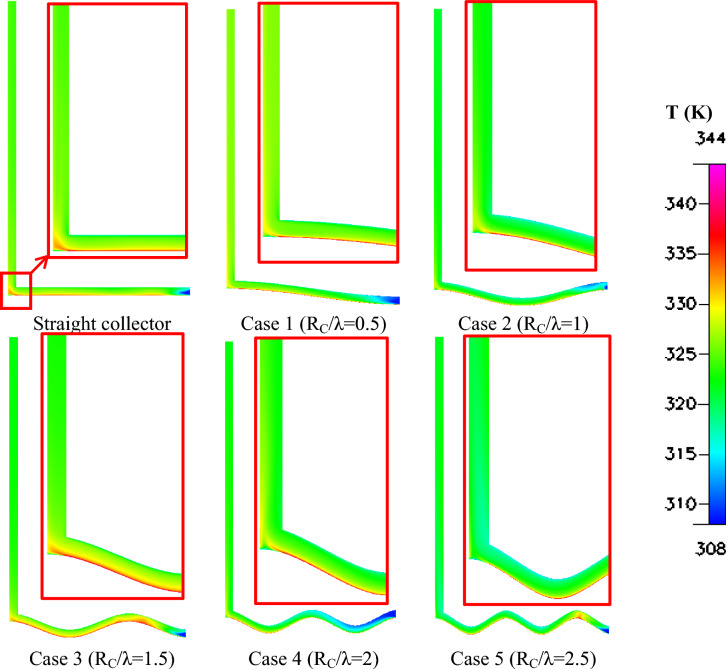
Different shapes of temperature contours (K) of the wavy collector solar chimney compared to the straight collector.

The thermal behavior of the system is illustrated in Fig. 12, which shows the temperature distribution within the collector region. In the straight collector configuration, the temperature gradient develops smoothly from the inlet toward the chimney base due to gradual heating of the airflow along the collector floor. With the introduction of surface waviness, the airflow remains in contact with the heated surface for a longer period, which increases heat transfer from the ground to the air. Consequently, higher temperature regions develop near the collector bottom surface, particularly for Rc/λ = 1 and Rc/λ = 1.5. This enhanced heating increases the density difference between the hot air inside the collector and the cooler ambient air, thereby strengthening the buoyancy forces responsible for the chimney draft.

**Fig. 13:**
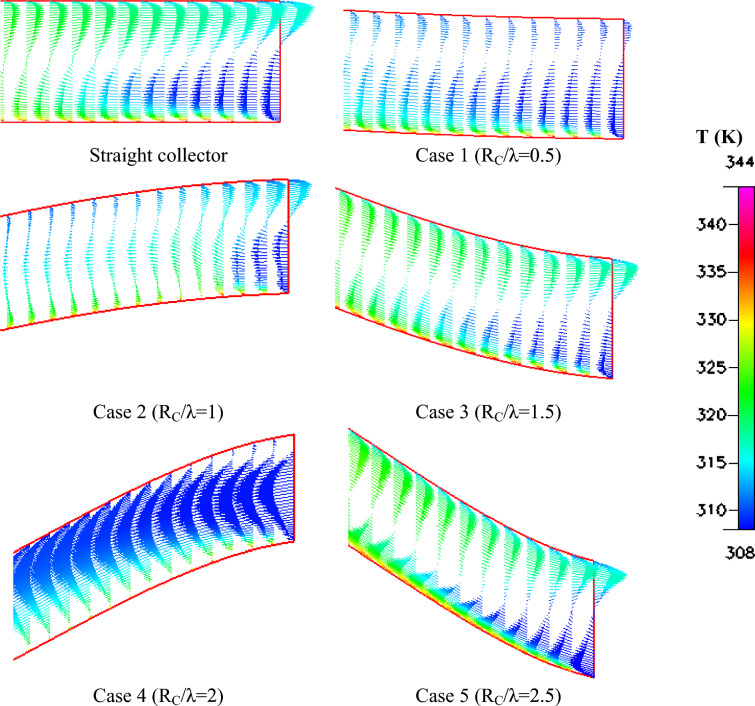
Velocity vectors at the collector entrance of different shapes of wavy collector solar chimney compared to the straight collector.

The velocity vector fields presented in Fig. 13 provide additional insight into the flow structure inside the collector. Moderate waviness promotes small-scale vortical structures that improve mixing between the heated boundary layer and the core airflow. This mixing mechanism enhances the thermal energy transfer to the bulk air and contributes to higher airflow velocities. However, when the curvature becomes excessive, as observed in Rc/λ = 2.5, large recirculation zones begin to form near the collector inlet. These adverse flow structures increase aerodynamic resistance and reduce the effective intake of ambient air, leading to a reduction in the overall mass flow rate through the system.

**Fig. 14:**
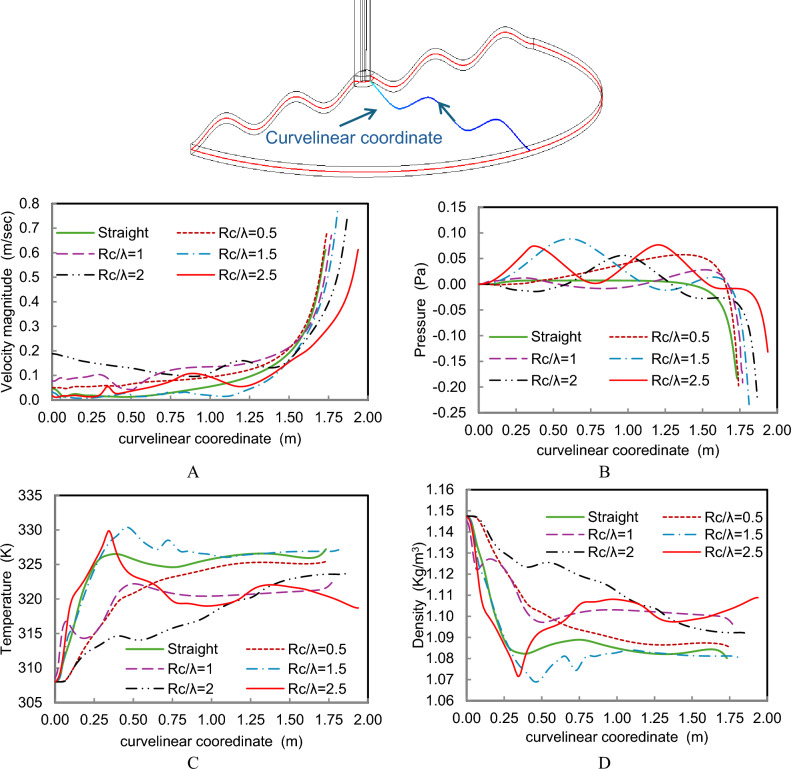
(**A**) velocity magnitude (m/s) (**B**) Pressure (Pa) **C**) Temperature (K) (**D**) Density (kg/m^3^), along the curvilinear coordinate of different wavy collector shapes compared to a straight collector.

A more detailed evaluation of the thermo-fluid development inside the collector is presented in Fig. 14, which shows the variation of velocity, pressure, temperature, and density along the curvilinear flow path from the collector inlet to the chimney outlet. The results indicate that wavy geometries delay the initial acceleration of the airflow but subsequently produce a sharper velocity increase near the chimney region. This behavior suggests improved momentum transport within the collector. In addition, the temperature distribution shows localized hot air regions for Rc/λ = 1.5, which enhance the buoyancy effect and contribute to stronger airflow through the chimney.

**Fig. 15:**
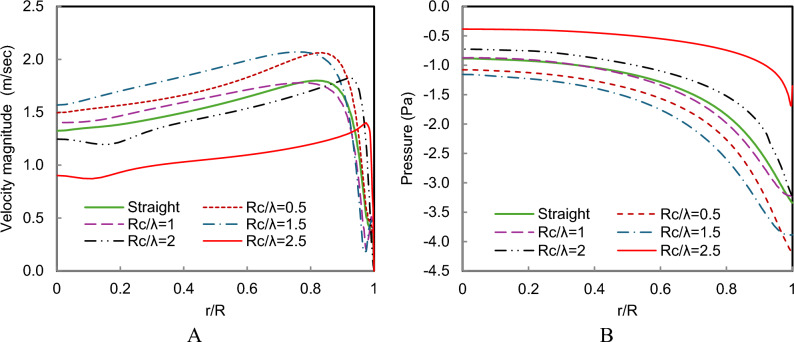
(**A**) velocity profile, (**B**) Pressure profiles at the chimney entrance of different wavy collector shapes compared to a straight collector.

The radial profiles at the chimney entrance, shown in Fig. 15, further highlight the influence of collector geometry on the system performance. For most wavy configurations, the velocity magnitude near the chimney entrance exceeds that of the straight collector, confirming the aerodynamic advantage of the modified collector design. The Rc/λ = 1.5 configuration achieves the highest peak velocity, reaching approximately 2.2 m/s, which corresponds to the strongest chimney draft among the tested cases. Similarly, the pressure profiles indicate that the negative pressure magnitude increases with curvature up to Rc/λ = 1.5, after which it decreases due to the adverse flow effects introduced by excessive waviness.

Table 3 provides the performance analysis of various wavy collector geometries in comparison to the straight collector configuration, revealing important insights into their effect on flow dynamics at the chimney entrance of an SCPP. The results focus on how changes in the collector shape, characterized by the ratio Rc /λ, influence the path length, velocity, negative pressure, and temperature at the chimney inlet. As the wavy profile becomes more pronounced (i.e., increasing Rc /λ), the path length of the airflow increases, reaching up to 20.6 cm at Rc/λ = 2.5, compared to the straight collector. This extended surface area has a direct impact on airflow characteristics. For moderate values like Rc/λ = 1.5, a significant velocity increase of 29.13% and negative pressure improvement of 27.56% are observed, indicating optimal aerodynamic performance. These values suggest that this geometry promotes more efficient natural convection, enhancing the pressure difference driving the airflow.

**Table 3:** Effect of different wavy collector shapes on chimney entrance properties.

Case	Rc/λ	Increment in path length (cm)	Percentage (%) increment at chimney entrance		
			Velocity	Negative Pressure	Temperature
	Straight	–	–	–	–
1	0.5	1.0	11.82	7.59	− 0.51
2	1	3.67	9.64	4.52	− 1.48
3	1.5	8.03	29.13	27.56	− 0.03
4	2	13.75	21.63	25.7	− 1.06
5	2.5	20.6	0.09	− 28.15	− 2.6

On the other hand, smaller curvatures (e.g., Rc /λ = 0.5) also improve velocity and suction at the entrance but to a lesser extent (11.82% and 7.59%, respectively). This indicates that although these designs contribute positively, their enhancements are not as impactful as those seen with moderate waviness. Interestingly, the temperature at the chimney entrance slightly decreases across all wavy configurations, with the most notable drop being -2.6% at Rc/λ = 2.5. This shows that while increasing surface area can improve heat transfer and airflow, it must be optimized to avoid introducing adverse aerodynamic effects.

**Fig. 16:**
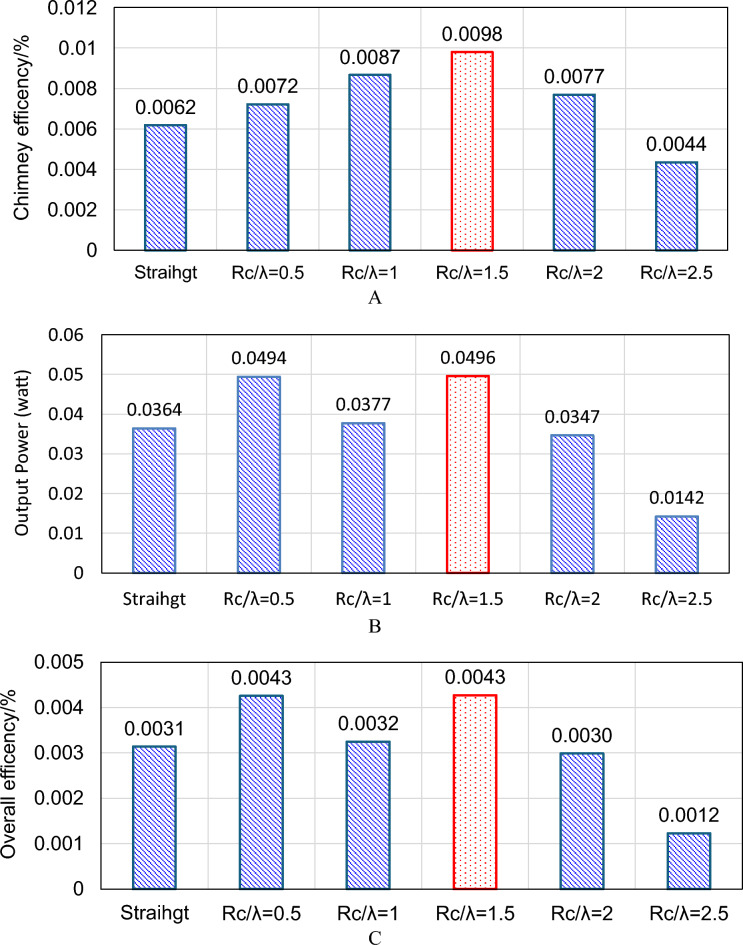
Effect of different wavy collector shapes on (**A**) Chimney efficiency (**B**) Output power (watt) (**C**) Overall efficiency.

Figure 16 illustrates the influence of different wavy collector geometries, represented by the ratio Rc/λ, on the chimney efficiency, output power, and overall system efficiency compared with the straight collector configuration. As shown in Fig. 16A, the chimney efficiency increases when the collector geometry changes from the straight configuration (0.0062%) to the wavy shapes. The efficiency rises gradually to 0.0072% at Rc/λ = 0.5 and 0.0087% at Rc/λ = 1, reaching a maximum value of 0.0098% at Rc/λ = 1.5. Beyond this point, the efficiency decreases to 0.0077% and 0.0044% for Rc/λ = 2 and Rc/λ = 2.5, respectively. This indicates that moderate wave amplitudes enhance airflow acceleration inside the chimney, while excessive waviness increases flow resistance and reduces performance.

A similar trend is observed for the output power in Fig. 16B. The output power increases from 0.0364 W for the straight collector to 0.0494 W at Rc/λ = 0.5 and reaches its peak value of 0.0496 W at Rc/λ = 1.5. After this optimum point, the power output decreases significantly, reaching 0.0142 W at Rc/λ = 2.5. This behavior confirms that an appropriate wavy configuration enhances heat transfer and buoyancy-driven airflow, which improves the power generation capability. The overall efficiency, shown in Fig. 16C, follows the same pattern. The efficiency increases from 0.0031% for the straight collector to 0.0043% at Rc/λ = 0.5 and reaches its maximum value of 0.0043% at Rc/λ = 1.5 before decreasing again for higher ratios. Overall, the results demonstrate that the Rc/λ = 1.5 configuration provides the optimal collector geometry among the tested cases, yielding the highest chimney efficiency, output power, and overall efficiency.

### Effect of wave amplitude, A, on flow field

After identifying the curvature ratio Rc/λ = 1.5 as the most favorable configuration for enhancing airflow and pressure drop at the chimney entrance, a second parametric study was conducted to investigate the influence of the wave amplitude (A) on the thermo-fluid behavior of the solar chimney system. The amplitude controls the vertical displacement of the collector surface and therefore directly affects the local flow passage geometry, heat transfer interaction, and buoyancy-driven airflow development inside the collector.

**Fig. 17:**
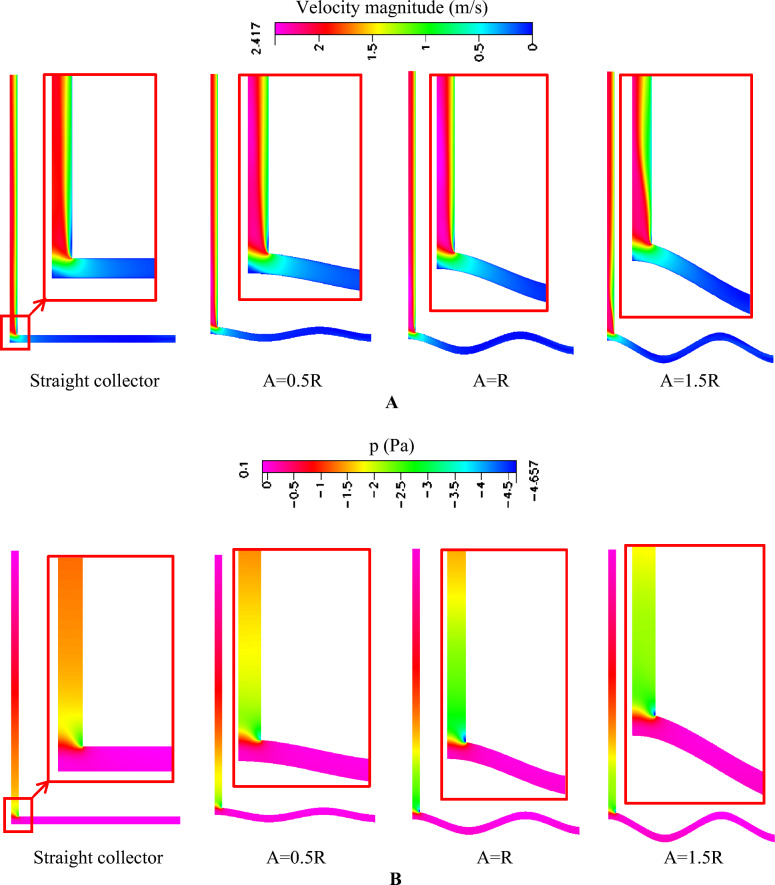
Effect of wave amplitude with Rc/λ = 1.5 on solar chimney (**A**) Velocity contours (m/s) (**B**) pressure contours (Pa).

Figure 17 illustrates the velocity and pressure contours for different wave amplitudes, including the straight collector configuration used as the baseline case. The results clearly indicate that introducing surface waviness increases the acceleration of the airflow beneath the collector roof. This enhancement occurs because the wavy surface modifies the boundary layer development and increases the effective interaction between the heated ground surface and the flowing air. As the wave amplitude increases, the airflow is forced to follow a more curved trajectory, which promotes local mixing and increases the convective heat transfer rate.

For A = 0.5R, the velocity contours show a moderate increase in airflow acceleration compared with the straight collector case. The flow remains relatively smooth with only minor disturbances in the velocity field, indicating that the waviness is sufficient to enhance heat transfer while maintaining a stable flow structure. When the amplitude is increased to A = R, the acceleration becomes more pronounced, particularly near the collector outlet and the chimney entrance. This configuration produces the highest velocity magnitude within the collector, indicating that the interaction between thermal buoyancy and geometric flow guidance is maximized.

The pressure contours in Fig. 17B support these observations. Increasing the wave amplitude intensifies the negative pressure region near the chimney base, which strengthens the suction effect responsible for drawing air through the collector. The most pronounced pressure reduction occurs for A = R, indicating that this amplitude produces the strongest buoyancy-driven draft in the system. This behavior results from the combined effect of increased air heating and improved flow guidance toward the chimney.

However, when the amplitude is further increased to A = 1.5R, the improvement in airflow acceleration becomes less significant, and a slight reduction in velocity can be observed in certain regions. This behavior suggests that excessive amplitude begins to introduce aerodynamic penalties. The larger geometric undulations create longer flow paths and may generate localized recirculation zones, which increase viscous resistance and partially offset the benefits of enhanced heat transfer.

**Fig. 18:**
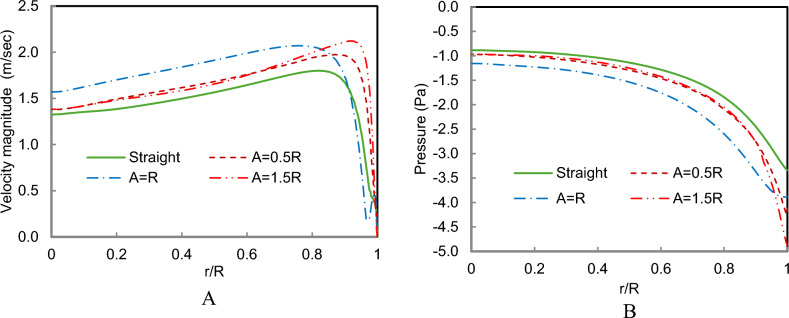
(**A**) velocity profile, (**B**) Pressure profiles at the chimney entrance of different collector wave amplitudes with Rc/λ = 1.5 compared to a straight collector.

The radial velocity and pressure profiles at the chimney entrance, shown in Fig. 18, provide further insight into the influence of amplitude on system performance. Figure 18A shows that the peak velocity increases progressively from the straight collector case to A = 0.5R and reaches its maximum value for A = R. This indicates that moderate waviness significantly enhances airflow acceleration inside the collector and strengthens the updraft in the chimney. However, for A = 1.5R, the velocity profile begins to decline near the collector edge, suggesting that excessive amplitude introduces additional flow resistance and reduces the effectiveness of the chimney draft.

A similar trend is observed in the pressure distribution shown in Fig. 18B. The magnitude of the negative pressure increases as the amplitude increases up to A = R, indicating stronger suction and enhanced buoyancy-driven airflow. Beyond this amplitude, the pressure reduction becomes less pronounced, confirming that very large surface undulations can disturb the pressure field and limit further performance improvements.

A comparative evaluation with previously published studies on solar chimney power plant (SCPP) performance enhancement indicates that the aerodynamic and thermal trends observed in the present work are consistent with the general behavior reported in the literature. Several researchers have demonstrated that modifying the collector geometry can significantly improve buoyancy-driven airflow by enhancing heat transfer between the absorber surface and the flowing air. For instance, stepped or structured absorber configurations were shown to increase airflow velocity and pressure drop by promoting stronger thermal interaction and flow mixing inside the collector region^7^. Similarly, the introduction of vortex generators and other surface disturbances has been reported to intensify local turbulence and improve the thermo-hydraulic performance of solar air heating systems and solar chimney collectors through enhanced convective heat transfer^8,9^. The results obtained in the present study follow a comparable trend: moderate surface waviness improves airflow acceleration and increases negative pressure near the chimney entrance, due to stronger buoyancy effects and improved flow guidance. However, the present results also reveal that excessive geometric waviness may introduce additional flow resistance and localized recirculation zones, which can partially offset the aerodynamic benefits. This observation is in agreement with previous studies indicating that an optimal level of geometric modification is required to balance heat transfer enhancement and aerodynamic losses in solar chimney collectors^5,6^. Overall, the current findings support the concept that carefully designed passive geometric modifications can significantly improve the thermo-fluid performance of SCPP systems while maintaining structural simplicity and operational reliability.

The present study provides useful insights into the aerodynamic and thermal behavior of a solar chimney power plant with a wavy collector configuration; however, several limitations should be acknowledged. The numerical analysis was conducted under steady-state conditions, assuming a constant heat flux at the collector base to represent solar radiation, thereby simplifying the actual transient variation of solar irradiance throughout the day and across different seasons. In addition, the model considers idealized boundary conditions and neglects some real environmental influences such as wind effects, ambient temperature fluctuations, and ground thermal storage characteristics. The optical properties of the collector cover were also assumed constant, whereas in practical systems, they may vary with material properties and operating conditions. Furthermore, the study focuses on a scaled configuration intended to evaluate the influence of the proposed wavy geometry rather than performing a full-scale plant optimization. Therefore, future work should include transient simulations, seasonal solar radiation data, and experimental validation for larger-scale systems to further assess the practical applicability and performance of the proposed collector design.

## Conclusion

This study aimed to evaluate the impact of applying wavy geometries with varying wavelength and wave amplitudes on the collector roof of an SCPP, focusing on aerodynamic performance enhancements. Through comprehensive CFD simulations, flow characteristics such as velocity distribution, pressure drop, and velocity vector behavior were analyzed for both straight and wavy collector configurations. The results were validated against experimental data and quantitatively assessed using error metrics, including RMSE and MAE. The following conclusions summarize the key findings derived from the comparative analysis:

- The computational results showed good agreement with available experimental data Nasraoui et al., with low error metrics RMSE and MAE, validating the reliability of the simulation setup.
- Compared to the straight collector configuration, introducing waviness in the collector improves airflow velocity and reduces pressure, thereby enhancing the overall efficiency of the solar chimney system.
- A curvature ratio of Rc /λ = 1.5 showed the best overall performance, increasing the air intake and velocity inside the collector while maintaining structural feasibility and flow uniformity.
- When the curvature was increased beyond Rc /λ = 2.5, flow separation and adverse pressure gradients occurred at the collector inlet, leading to backflow zones and reduced air intake, thus degrading system performance.
- Amplitude has a nonlinear impact on system performance, as among all tested amplitudes, A = R provided the most favorable results in terms of air velocity and pressure drop. However, further increasing the amplitude to A = 1.5R did not yield significant additional gains and showed signs of localized velocity decay near the outlet.
- Both wavy curvature and wave amplitude modifications contributed to stronger pressure gradients and higher velocity distributions across the collector radius, especially in the region near the chimney entrance.
- Most of the flow acceleration and beneficial effects of waviness occur near the outer radius r/R > 0.8, indicating that geometric modifications have their greatest influence close to the chimney entrance.
- Velocity vector and contour plots showed that the wavy configurations introduced complex vortex structures and stronger shear layers near the wavy walls, which enhanced mixing and air induction.
- Both curvature and amplitude improvements should be applied within optimal ranges, Rc/λ = 1.5, A = R, to avoid performance degradation due to flow detachment or instability.
- The study provides clear evidence that passive geometric enhancements, particularly wavy profiles with tuned curvature and amplitude, can significantly improve the aerodynamic performance and should be considered in future solar chimney designs for renewable energy generation.

## Future work

- Investigate the time-dependent behavior of the solar chimney system under varying solar radiation and to better understand the dynamic response of the buoyancy-driven airflow.
- Perform a year round performance analysis using long term solar irradiance data to evaluate seasonal variations and provide a more comprehensive assessment of the annual performance of the proposed SCPP design.
- Conduct a detailed second-law thermodynamic analysis to quantify entropy generation and viscous dissipation, assessing the trade-off between enhanced mixing and associated irreversibilities in the wavy solar chimney design.
- Perform a comprehensive techno-economic analysis to evaluate the practical feasibility, including construction costs, material requirements, and expected power output, of the proposed wavy collector design at utility scale.
- The present analysis will be extended to large-scale solar chimney systems under varying climatic conditions, including different solar irradiance levels, ambient temperatures, and wind effects, in order to evaluate the robustness and practical applicability of the proposed wavy collector design.

## Data Availability

The data presented in this study is available on request from the corresponding author.

## Abbreviations

*A*: Peak amplitude of curved collector (m)
*G*: Solar irradiation ($$\mathrm{W}/{\mathrm{m}}^{2}$$)
*H*_**C**_: Collector height (m)
*H*_ch_: Chimney height (m)
$\dot{m}$: Chimney mass flow rate ($\mathrm{Kg}/{\mathrm{m}}^{3}$)
*R*: Radius of solar chimney (m)
*Rc*: Radius of collector (m)
*r*: Cylindrical coordinate variable (m)
*T*: Temperature (K)
*ΔT*: Chimney temperature difference (K)
*u*_*r*_, *u*_*θ*_, u_*z*_: Cylindrical velocity components (m/s)
*V*: Local velocity magnitude (m/s)

## Greek symbols

*θ*: Angle, cylindrical coordinate variable (degree)
$\Phi$: Viscous dissipation rate (kg/m s^3^)
*µ*: Dynamic viscosity (kg/m s)
$$\rho$$: Density (kg/m^3^)
${\rho }_{0}$: Reference density (kg/m^3^)
*β*: Air thermal expansion coefficient (K^−^^1^)
${\eta }_{c}$: Efficiency of collector
${\eta }_{ch}$: Efficiency of chimney
${\eta }_{o}$: System overall efficiency
